# Analyse des Hautmikrobioms eines an junktionaler Epidermolysis bullosa erkrankten Patienten nach Behandlung mit genetisch modifizierten Stammzellen

**DOI:** 10.1111/ddg.15776_g

**Published:** 2025-09-15

**Authors:** Alexander Dermietzel, Burcu Tosun, Mathilde Nguyen, Kai Wessel, Luise Rauer, Avidan U. Neumann, Tobias Hirsch, CK‐CARE study group, Claudia Traidl‐Hoffmann, Matthias Reiger, Claudia Hülpüsch, Maximilian Kueckelhaus

**Affiliations:** ^1^ Abteilung für Plastische Chirurgie Universitätsklinikum Münster Münster Deutschland; ^2^ Abteilung für Plastische Rekonstruktive und Ästhetische Chirurgie Handchirurgie Fachklinik Hornheide Münster Deutschland; ^3^ Abteilung für Plastische und Rekonstruktive Chirurgie Institut für Muskuloskelettale Medizin Universität Münster Münster Deutschland; ^4^ Abteilung für Plastische Rekonstruktive und Ästhetische Chirurgie Handchirurgie Rotenburg Deutschland; ^5^ Institut für Umweltmedizin und Integrative Gesundheit Medizinische Fakultät Universität Augsburg Augsburg Deutschland; ^6^ Institut für Umweltmedizin Helmholtz München Augsburg Deutschland; ^7^ CK‐CARE Christine Kühne Center for Allergy Research and Education Davos Schweiz

**Keywords:** Hautmikrobiom, Junktionale Epidermolysis bullosa, Staphylococcus aureus, Junctional Epidermolysis Bullosa, Skin microbiome, Staphylococcus aureus

## Abstract

**Hintergrund und Ziel:**

Die junktionale Epidermolysis bullosa (JEB) ist eine Unterform der Epidermolysis bullosa, einer Erkrankung, die durch eine Mutation im *LAMB3*‐Gen verursacht wird. Wir behandelten einen Patienten mit JEB mit genetisch korrigierten autologen epidermalen Kulturen unter Verwendung eines retroviralen Vektors, der die funktionelle Gensequenz des *LAMB3*‐Gens enthält. Ziel dieser Studie war es, das Hautmikrobiom des Patienten, insbesondere der transgenen Haut, zu analysieren und mit dem Hautmikrobiom gesunder Kontrollpersonen sowie Patienten mit atopischer Dermatitis und bekannter mikrobieller Dysbiose zu vergleichen.

**Patienten und Methodik:**

Die Analyse des Hautmikrobioms wurde 72 Monate nach kombinierter Gen‐ und Stammzelltherapie bei einem JEB‐Patienten durchgeführt. Hautabstriche von altersentsprechenden gesunden Kontrollpersonen und Patienten mit atopischer Dermatitis wurden aus der ProRaD‐Studie von CK‐CARE einbezogen.

**Ergebnisse:**

Die transgene Haut wies vergleichbar hohe relative und absolute Häufigkeiten von *Staphylococcus (S.) aureus* auf wie die blasenbildende und nicht‐blasenbildende Haut des JEB‐Patienten, während die Gesamtbakterienlast geringer war. In der blasenbildenden Haut war die erhöhte Bakterienlast durch *S. aureus* bedingt.

**Schlussfolgerungen:**

Unsere Untersuchung bestätigt eine einzigartige Zusammensetzung des Mikrobioms bei JEB, die durch *S.‐aureus*‐getriebene bakterielle Überwucherung charakterisiert ist. Die Dysbiose wurde in transgenen, nicht‐blasenbildenden Hautbereichen nicht rückgängig gemacht. Allerdings zeigt die transgene Haut Stabilität in einer Umgebung mit bakterieller Dysbiose.

## EINLEITUNG

Die junktionale Epidermolysis bullosa (JEB) ist eine Unterform der Epidermolysis‐bullosa (EB)‐Gruppe und eine schwere genetische Erkrankung, die durch eine Mutation im *LAMB3*‐Gen (kodiert die β3‐Kette von Laminin 332) verursacht wird.^1^, ^2^, ^3^, ^4^ Betroffene Patienten leiden unter Blasenbildung der Haut, die durch geringfügigen mechanischen Stress ausgelöst wird. Schwere Blasenbildung führt zu Narbenbildung, Infektionen und einem fortschreitenden Krankheitsverlauf bis hin zu frühem Tod. Über 40 % der Patienten sterben vor Erreichen des Erwachsenenalters.^5^, ^6^, ^7^

Bis 2015 existierte keine definitive Behandlung für vererbte JEB. Unser Forschungsteam behandelte einen jungen Patienten mit JEB, dessen Epidermis zu etwa 80 % zerstört war. Nachdem alle etablierten Therapien fehlgeschlagen waren, entschieden wir uns für einen experimentellen Ansatz und transplantierten Haut, die aus genetisch modifizierten Stammzellen hergestellt wurde, auf die Wundflächen. Die Stammzellen wurden über eine Hautbiopsie entnommen und mithilfe eines retroviralen Vektors, der die funktionelle Gensequenz des *LAMB3*‐Gens enthält, transduziert.^8^, ^9^, ^10^

Vor der Transplantation bestand trotz regelmäßiger Antibiotikabehandlung eine Superinfektion mit *Staphylococcus (S.) aureus*. Die Infektion wurde zunehmend schwerer, bis der Patient zum Zeitpunkt der Transplantation eine schwere Sepsis mit beginnendem Organversagen erlitt. Die Transplantation der genetisch transduzierten Haut war ein Erfolg. Unser Patient erholte sich und wurde aus dem Krankenhaus entlassen. Ein 5‐Jahres‐Nachuntersuchungszeitraum zeigte eine langfristige Stabilität der gesamten transgenen Epidermis ohne erneute Blasenbildung in den transplantierten Bereichen. Diese Ergebnisse wurden in früheren Studien unseres Teams veröffentlicht.^8^, ^11^, ^12^

Die menschliche Haut ist nicht nur das größte Organ und Schutz vor Umwelteinflüssen, sondern wird selbst durch ein kutanes Mikrobiom geschützt. Dieses dient als zusätzliche Barriere zur physischen und chemischen Schutzbarriere gegenüber Umwelteinflüssen und schützt die Haut vor Krankheiten.^13^, ^14^ Es handelt sich um eine komplexe und dynamische Gemeinschaft von Bakterien, Pilzen und anderen Mikroorganismen, die auf der Hautoberfläche leben. Das Mikrobiom spielt eine wichtige Rolle bei der Erhaltung der Hautgesundheit, dem Schutz vor Krankheitserregern und der Regulierung des Immunsystems der Haut und ist für jeden Menschen einzigartig. Es kann durch Faktoren wie Alter, Geschlecht, Lebensstil, Umwelt, Krankheiten und medizinische Behandlungen beeinflusst werden.^15^

Eine Dysbiose des Hautmikrobioms wird mit Hauterkrankungen wie der atopischen Dermatitis (AD) in Verbindung gebracht, bei der eine erhöhte Häufigkeit von *S. aureus* die Krankheitsausprägung beispielsweise durch Toxinproduktion verstärken kann.^16^, ^17^, ^18^, ^19^, ^20^

Das genaue Mikrobiom bei EB wurde bisher nicht umfassend untersucht. Studien zeigen jedoch, dass Personen mit EB ein verändertes Mikrobiom im Vergleich zu gesunden Individuen aufweisen, mit einer Überhäufigkeit von *S. aureus* und einer insgesamt geringeren Diversität der Hautmikroben.^21^, ^22^

Aufgrund dieser vorangegangenen Ergebnisse entschied sich unser Forschungsteam, intraindividuelle Veränderungen des Hautmikrobioms bei unserem mit genetisch korrigierten autologen epidermalen Kulturen behandelten Patienten weiter zu untersuchen und das Hautmikrobiom mit gesunden Kontrollen und AD‐Patienten zu vergleichen.

## ZIELSETZUNG

Das Ziel dieser Forschung war es, das Hautmikrobiom eines mit genetisch korrigierten autologen epidermalen Kulturen behandelten JEB‐Patienten zu analysieren. Von besonderem Interesse war die mikrobielle Zusammensetzung der transgenen Hautbereiche im Vergleich zu den umgebenden blasenbildenden und nicht‐blasenbildenden Hautbereichen des JEB‐Patienten 72 Monate nach der kombinierten Gen‐ und Stammzelltherapie.

Zusätzlich wurde das Hautmikrobiom dieses JEB‐Patienten mit der mikrobiellen Zusammensetzung der Haut gesunder Kontrollpersonen und AD‐Patienten verglichen.

## PATIENTEN UND METHODIK

Alle Studienmethoden entsprachen der *Deklaration von Helsinki*. Die ethische Zustimmung für diese Studie wurde von der *Ethikkommission der Universität Münster* erteilt (Referenz: 2020‐804‐f‐S). Daten von AD‐Patienten wurden aus der Prospektive Längsschnittstudie zur Untersuchung der Remissionsphase bei Patienten mit atopischer Dermatitis (AD). Diese Studie wurde von den jeweiligen lokalen Ethikkommissionen in Zürich, Schweiz (BASEC 2016‐00301, ClinicalTrials.gov Identifier: NCT04240522), der Technischen Universität München (112/16S) und Bonn (ProRaD 232/15) genehmigt. Die ProRaD‐Studie untersucht Biomarker und das kutane Mikrobiom von AD‐Patienten in Remission in einem longitudinalen, prospektiven Setting.^23^

### Studienpopulation

**ABBILDUNG 1:**
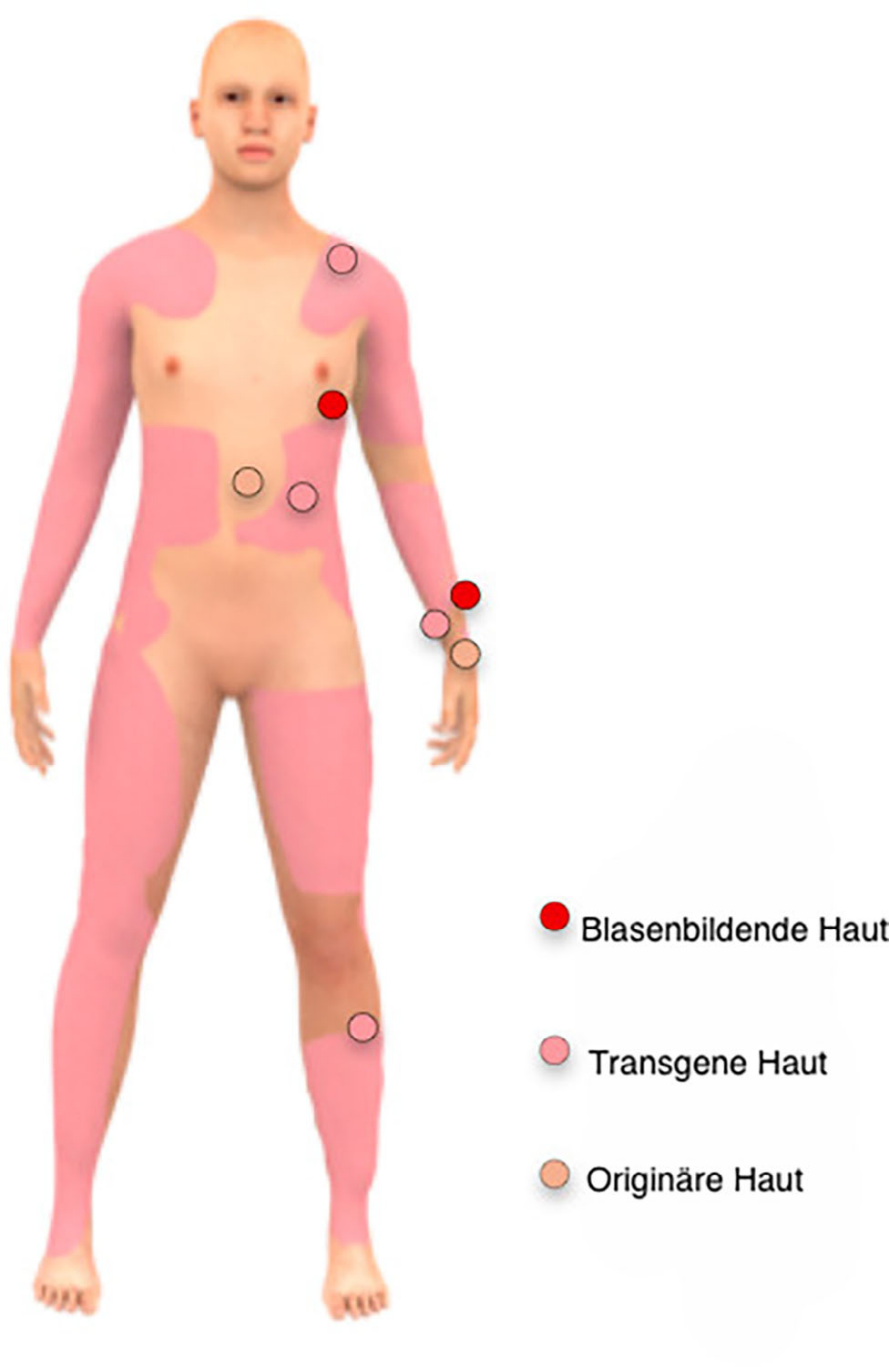
Hautmerkmale des JEB‐Patienten. Blasenbildende Haut, nicht‐blasenbildende Haut und transgene Hautareale des JEB‐Patienten. Unterarm, Handgelenk, Unterschenkel, Thorax, Abdomen und Schulter wurden für die Mikrobiomanalyse beprobt.

Die Analyse des Hautmikrobioms wurde 72 Monate nach der kombinierten Gen‐ und Stammzelltherapie bei einem JEB‐Patienten durchgeführt. Hautproben wurden durch Abstriche von blasenbildender, nicht‐blasenbildender und transgener Haut für die Mikrobiomanalyse entnommen. Hautabstriche wurden vom Unterarm, Handgelenk, Unterschenkel, Thorax, Bauch und Schulter entnommen, wie in Abbildung 1 gezeigt. Pro Hautareal wurden Replikate entnommen. Zudem wurden parallel zum JEB‐Patienten drei Abstriche von einer gesunden Person genommen, um die Daten mit einem weiteren Datensatz gesunder Kontrollpersonen zusammenführen und die Mikrobiom‐Befunde des JEB‐Patienten besser einordnen zu können.

Als altersentsprechende Kontrollen wurden vier gesunde Kinder und zehn gesunde Erwachsene aus der ProRaD‐Studienpopulation einbezogen. Um die mikrobielle Zusammensetzung bei JEB mit einer Erkrankung zu vergleichen, die durch eine bekannte mikrobielle Dysbiose gekennzeichnet ist, wurden zusätzliche Hautproben aus der Ellenbeuge – einem typischerweise betroffenen Areal bei Patienten mit atopischer Dermatitis – aus der ProRaD‐Studie einbezogen, wie in Tabelle 1 im Detail beschrieben (5 nicht‐läsionale Proben von Kindern, 5 läsionale Proben von Kindern, 10 nicht‐läsionale Proben von Erwachsenen und 10 läsionale Proben von Erwachsenen).

**TABELLE 1:** Übersicht der Proben. Die Anzahl der Proben pro Gesundheits‐ und Hautstatus ist in der Tabelle zusammengefasst.

Gesundheitsstatus	Hautstatus	Altersgruppe	Entnahmestelle	Studienherkunft	Verfügbare Proben pro Analyse Gesamt (qPCR, NGS)
JEB	Ebb	Kind	Unterarm, Brust	Münster	6 (6, 3)
EB	Ebnb	Kind	Hand, Handgelenk, Bauch	Münster	6 (6, 5)
JEB	T	Kind	Unterarm, Unterschenkel, Bauch, Schulter	Münster	8 (8, 8)
HE	NL	Erwachsener	Brust, Arm, Schulter	Münster	3 (3, 3)
HE	NL	Kind	Ellenbeuge	ProRaD	4 (4, 4)
HE	NL	Erwachsener	Ellenbeuge	ProRaD	10 (10, 10)
AD	NL	Kind	Ellenbeuge	ProRaD	5 (5, 5)
AD	LS	Kind	Ellenbeuge	ProRaD	5 (5, 5)
AD	NL	Erwachsener	Ellenbeuge	ProRaD	10 (10, 10)
AD	LS	Erwachsener	Ellenbeuge	ProRaD	10 (10, 10)

*Abk*.: AD, atopische Dermatitis; Ebb, junktionale Epidermolysis bullosa, blasenbildende Haut; Ebnb, junktionale Epidermolysis bullosa, nicht‐blasenbildende Haut; JEB, junktionale Epidermolysis bullosa; HE, gesunde Kontrollpersonen; LS, läsionale Haut; NL, nicht‐läsionale Haut; NGS, *Next‐Generation Sequencing*; T, junktionale Epidermolysis bullosa, transgene Haut

### Datenanalyse

Probenentnahme: Für die 16S rRNA‐Gen‐Amplicon‐Sequenzierung wurden Proben aller Datensätze wie zuvor beschrieben vorbereitet.^24^ Hautabstriche (Sigma‐swab, MWE, Corsham, England) wurden entnommen und in 500 µL DNA‐Stabilisatorlösung (Stratec, Berlin, Deutschland) gelagert.

DNA‐Extraktion: Die DNA wurde mithilfe des QIAamp UCP Pathogen kit (Qiagen: Hilden, Deutschland) durchgeführt wie bereits publiziert.

### Vorbereitung Next‐Generation‐Sequencing (NGS)

Amplifikation: Die V1–V3‐Region des 16S rRNA‐Gens wurde mit den Primern 27F‐YM (5‐AGAGTTTGATYMTGGCTCAG‐3) und 534R (5‐ATTACCGCGGCTGCTGG‐3) amplifiziert. Barcodes wurden in einem zweiten PCR‐Schritt hinzugefügt.

Library Vorbereitung: AMPure XP Beads (Beckman Coulter, Fullerton, CA, USA) wurden zur Amplicon‐Reinigung verwendet. Die Proben wurden mit der Illumina MiSeq^®^‐Plattform (Illumina Inc., San Diego, CA, USA) unter Verwendung von 2 × 300 bp Paired‐End‐Reads (MiSeq® Reagent Kit v3, 600 Zyklen) sequenziert.

Bioinformatik: Das Denoising der Sequenzen wurde mit DADA2^25^ durchgeführt, und die Annotation erfolgte mit AnnotIEM.^26^ MicrobIEM^27^ wurde verwendet, um Kontaminanten, Singletons und Proben mit niedrigen Reads zu entfernen. Die in Tabelle 1 angegebenen Proben bestanden die Qualitätskontrolle.

### Zusammenführung von Datensätzen

Trotz der Verwendung derselben Protokolle für alle Proben wurden die Proben aus der Münster‐ und der ProRaD‐Studie in zwei verschiedenen Chargen sequenziert. Die JEB‐Proben sowie die ProRaD‐Proben, einschließlich der zusätzlichen Proben von AD‐Patienten und gesunden Kontrollpersonen, wurden in verschiedenen Sequenzierungsläufen verarbeitet. Um Verzerrungen, die durch die Sequenzierungsläufe und den Einfluss spärlicher Sequenzen entstehen, zu reduzieren, wurde die relative Häufigkeit jeder Spezies pro Probe berechnet. Die zehn häufigsten Spezies pro Sequenzierungslauf und Datensatz wurden extrahiert und zusammengeführt, was zu einem finalen Analysedatensatz mit 13 Spezies führte. Alle anderen wurden unter „andere“ zusammengefasst.

### Qualitätskontrolle der Zusammenführung von Datensätzen

Nach der Analyse des JEB‐Mikrobioms wurde ein weiterer Vergleich mit AD‐Patienten und altersentsprechenden gesunden Kontrollen durchgeführt. Um zu überprüfen, ob die gewählte Methode zur Zusammenführung von Datensätzen aus zwei Sequenzierungsläufen geeignet war, wurde das globale Mikrobiom zwischen den gesunden erwachsenen Individuen beider Datensätze verglichen, da dies die einzige Patientengruppe war, die in beiden Sequenzierungsläufen enthalten war. Da die Proben im globalen Mikrobiom eng gruppiert waren, wurde die Methode als geeignet angesehen, um die Proben des JEB‐Patienten mit denen von AD‐Patienten und altersentsprechenden gesunden Kontrollen zu vergleichen (Abbildung S1, Online‐Supplement).

### Quantifizierung via qPCR

Zur Quantifizierung der absoluten bakteriellen Last diente das 16S‐rRNA‐Gen als Marker. Für die Quantifizierung von *S. aureus* wurde das spezifische *S. aureus*‐Gen *nuc* verwendet. Die qPCR wurde mittels eines TaqMan‐Assays durchgeführt, unter Verwendung der folgenden Primer und Sonden:

*S. aureus*:

‐ *Forward‐Primer*: GTTGCTTAGTGTTAACTTTAGTTGTA
‐ *Reverse‐Primer*: AATGTCGCAGGTTCTTTATGTAATTT
‐ *Sonde*: FAM‐AAGTCTAAGTAGCTCAGCAAATGCA‐BHQ1^28^

*16S rRNA‐Gen‐Kopien*:

‐ *Forward‐Primer*: TGGAGCATGTGGTTTAATTCGA
‐ *Reverse‐Primer*: TGCGGGACTTAACCCAACA
‐ *Sonde*: Cy5‐CACGAGCTGACGACARCCATGCA‐BHQ2 (Eurogentec S.A., Seraing, Belgien)^29^

Die Reaktionen wurden in einem Endvolumen von 10 µL unter Verwendung des PerfeCTa Multiplex qPCR ToughMix (Quantabio, Beverly, MA, USA) mit einer Konzentration von 100 nM für jeden Primer und jede Sonde im Multiplex‐Setup durchgeführt. Nach einem zweiminütigen Denaturierungs‐/Aktivierungsschritt bei 95°C wurden 45 Zyklen mit einem Denaturierungsschritt von 15 Sekunden bei 95°C und einem Annealing‐/Elongation‐Schritt von 60 Sekunden bei 60°C in einem CFX384 Real Time System (Bio‐Rad Laboratories, Inc., Hercules, CA, USA) durchgeführt. Die Quantitätszyklen (Cqs) wurden als Mittelwert unabhängiger Triplikate bestimmt.

## ERGEBNISSE

### Hautmikrobiom der transgenen Haut des JEB‐Patienten

**ABBILDUNG 2:**
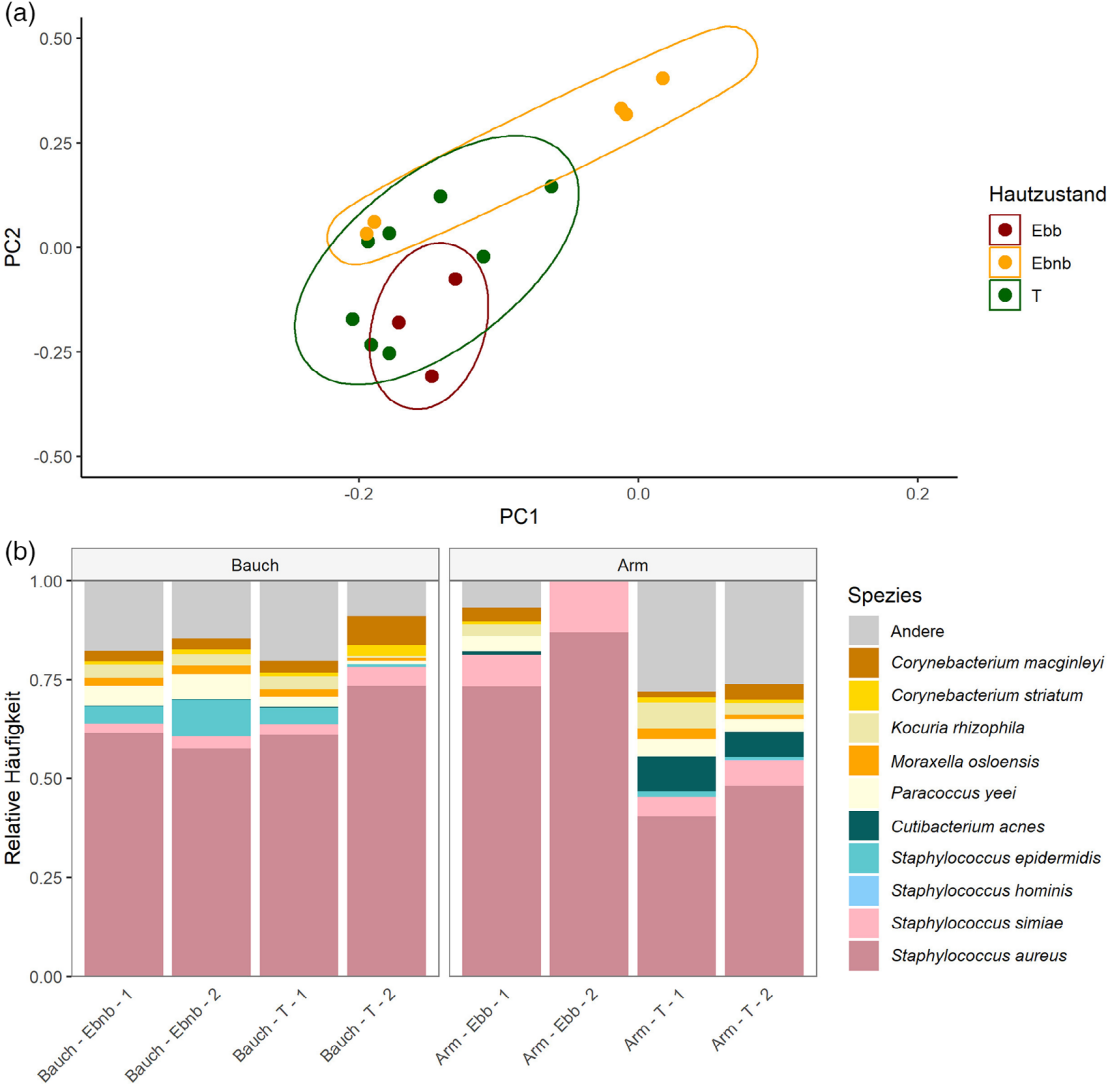
Das Hautmikrobiom des Transplantats ähnelt der umgebenden nicht‐blasenbildenden Haut des JEB‐Patienten. Das globale Mikrobiom der transgenen Haut gruppierte sich zwischen blasenbildender und nicht‐blasenbildender Haut. (a) Globales Hautmikrobiom dargestellt in einer Principal‐Coordinate‐Analyse (PCoA) basierend auf der Bray‐Curtis‐Distanz. (b) Taxonomische Zusammensetzung des Hautmikrobioms von blasenbildender, nicht‐blasenbildender und transgener Haut an Arm und Bauch. Die transgene Haut zeigt am Bauch ein ähnliches Mikrobiom wie die nicht‐blasenbildende Haut, während am Arm die relative Häufigkeit von *S. aureus* reduziert war. *Abk*.: Ebb, junktionale Epidermolysis bullosa, blasenbildende Haut; Ebnb, junktionale Epidermolysis bullosa, nicht‐blasenbildende Haut; T, junktionale Epidermolysis bullosa, transgene Haut

Das globale Hautmikrobiom des JEB‐Patienten unterschied sich je nach Hautstatus (blasenbildend, nicht‐blasenbildend und transgen). Die blasenbildende Haut (Ebb) und die nicht‐blasenbildende Haut (Ebnb) wiesen die deutlichsten Unterschiede im globalen Hautmikrobiom auf, während das transgene (T) Hautmikrobiom zwischen nicht‐blasenbildender und blasenbildender Haut lag, wie in Abbildung 2a dargestellt.

Die zehn häufigsten Spezies waren in allen Proben unabhängig vom Hautstatus und der Körperstelle vorhanden. Pro Körperstelle waren zwei Replikate verfügbar, die eine hohe Ähnlichkeit aufwiesen (Abbildung S2, Online‐Supplement). Generell war *S. aureus* die am häufigsten vorkommende Spezies. Die Zusammensetzung einzelner Proben variierte jedoch (Abbildung S2, Online‐Supplement).

Um den Einfluss der Körperstelle auf die Zusammensetzung des Hautmikrobioms auszuschließen, wurde die Zusammensetzung des Hautstatus nur innerhalb einer Körperstelle verglichen, an der verschiedene Hautstatus vorhanden waren (Arm, Bauch). Wie in Abbildung 2b dargestellt, zeigte der Arm eine höhere relative Häufigkeit von *S. aureus* in der blasenbildenden Haut im Vergleich zur benachbarten transgenen Haut, während im Bauch keine Unterschiede zwischen dem Hautmikrobiom der nicht‐blasenbildenden Haut und der transgenen Haut festgestellt wurden, die beide von *S. aureus* dominiert waren.

### *Staphylococcus‐aureus‐*bedingter Anstieg der bakteriellen Belastung in blasenbildender Haut

**ABBILDUNG 3:**
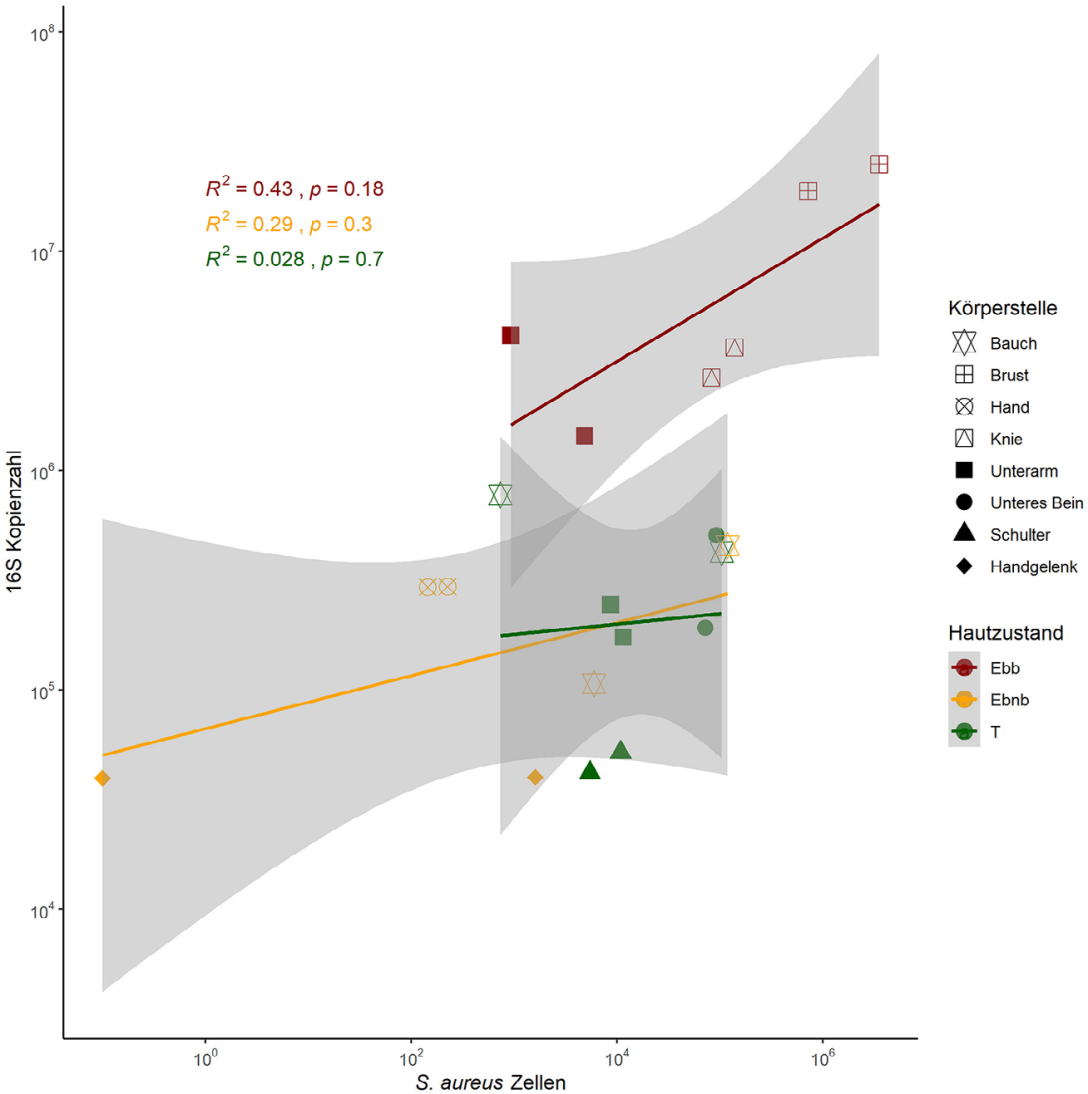
*Staphylococcus‐aureus*‐getriebene Bakterienüber besiedelung in der blasenbildenden Haut des JEB‐Patienten. qPCR des 16S‐rRNA‐Gens als Marker für die Bakterienzellzahl mit der höchsten Belastung in blasenbildender Haut. Die hohe Bakterienlast in blasenbildender Haut wurde durch die Zellzahl von *S. aureus* getrieben, was durch eine starke, aber nicht signifikante Korrelation gezeigt wurde. Die Spearman‐Korrelation wurde für jeden Probentyp durchgeführt. Die Entnahmestelle ist durch die Symbole dargestellt. *Abk*.: Ebb, junktionale Epidermolysis bullosa, blasenbildende Haut; Ebnb, junktionale Epidermolysis bullosa, nicht‐blasenbildende Haut; T, junktionale Epidermolysis bullosa, transgene Haut; Ebnb, 6 qPCR; Ebb, 6 qPCR; T, 8 qPCR.

In den blasenbildenden Hautbereichen des JEB‐Patienten waren sowohl die absoluten Zellzahlen von *S. aureus*, die mittels qPCR bestimmt wurden, als auch die 16S‐Kopienzahl als Stellvertreter für die Bakterienzellzahlen höher als in der nicht‐blasenbildenden und der transgenen Haut (Abbildungen S2–S4, Online‐Supplement). Die höhere bakterielle Belastung wurde durch *S.‐aureus*‐Zellen verursacht, wie in Abbildung 3 gezeigt. Obwohl die Häufigkeit von *S. aureus* in der transgenen Haut weiterhin hoch war, lagen die absoluten Bakterienzahlen unter denen der blasenbildenden Haut und auf dem gleichen Niveau wie in der nicht‐blasenbildenden Haut.

### Spezifisches Hautmikrobiom bei JEB

Ein Vergleich der zehn häufigsten Spezies des JEB‐Patienten mit gesunden Kontrollen und AD‐Patienten zeigte deutliche Unterschiede zwischen den Gruppen. Während die Haut des JEB‐Patienten stark von *S. aureus* dominiert wurde, beherbergte die Haut gesunder Kinder und Erwachsener eine Vielfalt von *Staphylococcus‐*, *Streptococcus‐* und *Cutibacterium*‐Arten. Interessanterweise waren bei der Haut des JEB‐Patienten spezifisch *Corynebacterium (C.) macginleyi*, *C. striatum* und *Kocuria rhizophila* vorhanden, während andere typische Kommensalen wie *S. epidermidis*, *S. hominis* und *Cutibacterium acnes* reduziert waren.

**ABBILDUNG 4:**
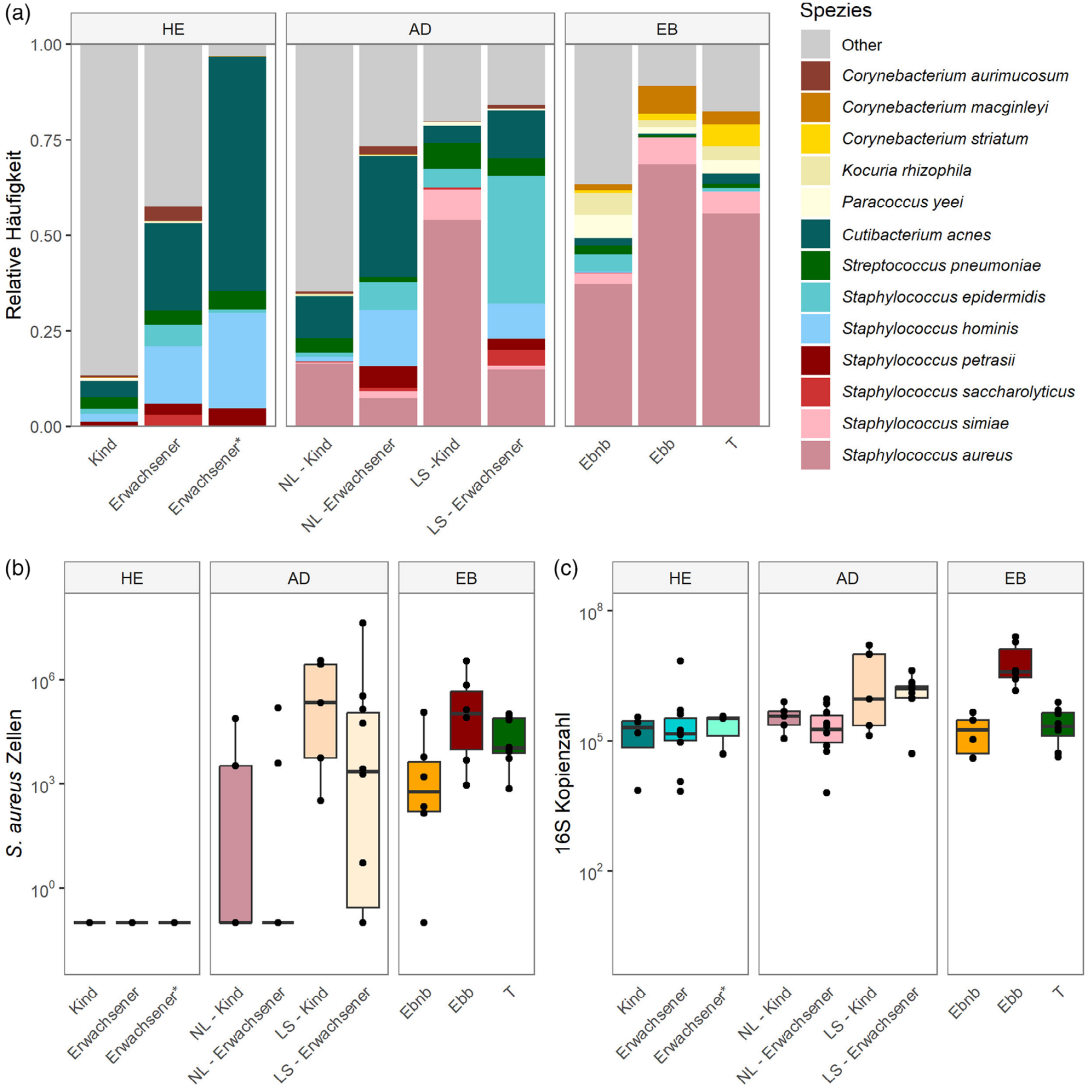
Das Hautmikrobiom des JEB‐Patienten unterscheidet sich deutlich von dem gesunder Kontrollpersonen und AD‐Patienten. (a) Die 13 häufigsten Spezies bei HE, AD und JEB unterscheiden sich. Auch der Hautstatus zeigt Unterschiede in der mikrobiellen Zusammensetzung. (b) Die über qPCR gemessenen absoluten Zellzahlen von *S. aureus* – insbesondere in blasenbildender Haut – entsprechen der *S.‐aureus*‐Belastung in AD‐läsionaler Haut. Die absoluten Bakterienzellzahlen – insbesondere in blasenbildender Haut – lagen auf ähnlichem Niveau wie in AD‐läsionalen Hautproben. *Abk*.:HE, gesunde Kontrollpersonen; AD, atopische Dermatitis; EB, junktionale Epidermolysis bullosa; NL, nicht‐läsionale Haut; LS, läsionale Haut; Ebb, junktionale Epidermolysis bullosa, blasenbildende Haut; Ebnb, junktionale Epidermolysis bullosa, nicht‐blasenbildende Haut; T, junktionale Epidermolysis bullosa, transgene Haut. HE Kind, 4 NGS, 4 qPCR; HE Erwachsene, 10 NGS, 10 qPCR; HE Erwachsene*, 3 NGS, 3 qPCR; AD NL Kind, 5 NGS, 5 qPCR; AD NL Erwachsene, 10 NGS, 10 qPCR; AD LS Kind, 5 NGS, 5 qPCR; AD LS Erwachsene, 10 NGS, 10 qPCR; Ebnb, 5 NGS, 6 qPCR; Ebb, 3 NGS, 6 qPCR; T, 8 NGS, 8 qPCR. *Gesunde erwachsene Person aus dem EB‐Datensatz.

Die relative Häufigkeit von *S. aureus* auf der Haut des JEB‐Patienten war sogar höher als in läsionalen Proben von AD‐Patienten (Abbildung 4). Darüber hinaus waren die absoluten Werte von *S. aureus* und bakteriellen Zellen ähnlich denen, die in läsionaler Haut von AD‐Patienten festgestellt wurden (Abbildung 4b,c). Obwohl in der transgenen Haut hohe Zellzahlen von *S. aureus* nachgewiesen wurden, war die absolute bakterielle Belastung ähnlich der in der nicht‐blasenbildenden Haut (Abbildung 4b,c).

## DISSKUSION UND LIMITATIONEN

In dieser Studie konnten wir zeigen, dass die nicht‐blasenbildende, die blasenbildende und die transgene Haut eines JEB‐Patienten eine Dysbiose in Bezug auf relative und absolute Zahlen von *S. aureus* aufweist. Die blasenbildende Haut zeigte eine durch *S. aureus* verursachte bakterielle Überwucherung. Das Hautmikrobiom des JEB‐Patienten unterschied sich deutlich von gesunden Kontrollen und AD‐Patienten.

Beim Vergleich gesunder Haut mit chronischen Wunden haben verschiedene Studien in der Vergangenheit eine reduzierte mikrobielle Diversität und eine hohe Häufigkeit von *S. aureus* in chronischen Wunden im Allgemeinen gezeigt.^21^, ^30^ Auch bei AD‐Patienten ist die Häufigkeit nützlicher Bakterien wie *S. epidermidis* reduziert, während andere Mikroorganismen wie *S. aureus*, *Pseudomonas aeruginosa* und *Malassezia*‐Arten vermehrt vorkommen.^15^, ^31^ Studien haben gezeigt, dass die relative und absolute Häufigkeit von *S. aureus* mit der Schwere der Erkrankung korreliert.^24^, ^32^

Nicht‐blasenbildende und verletzte Haut von Patienten mit rezessiver dystrophischer EB zeigt eine signifikant reduzierte Diversität und einen hohen Anteil von *S. aureus* sowohl in blasenbildender als auch in nicht‐blasenbildender Haut. Reimer‐Taschenbrecker et al. beobachteten eine Dominanz von *S. aureus*, die je nach Alter zunächst die verletzte/blasenbildende Haut und später die unverletzte/nicht‐blasenbildende Haut befällt.^1^

In solchen Fällen korrelieren die Schwere der Erkrankung und die Wundlast signifikant positiv mit der *S.‐aureus*‐Kolonisation, ähnlich wie bei anderen Hauterkrankungen wie AD. Ein Grund für die allgemeine Dysbiose und die Häufigkeit der *S.‐aureus*‐Kolonisation könnte der notwendige Einsatz von Wundverbänden bei blasenbildenden Bereichen sein, die oft überlappen und nicht‐blasenbildende Bereiche bedecken. Dies schafft ein günstiges Umfeld für die Verbreitung von *S. aureus* über die Hautoberfläche. Blasenbildende Hautbereiche sind zunächst betroffen, und Wundverbände können die bakterielle Ausbreitung fördern. Diese Hypothese wird durch die Ergebnisse von Horev et al. (2023) unterstützt, die eine höhere Häufigkeit von *S. aureus* in EB‐Wunden nach der Anwendung von Wundverbänden zeigten. Neunzig Tage nach der Wundbehandlung zeigten Kinder mit EB dystrophica und EB simplex eine signifikant höhere Inzidenz von *S. aureus* in den blasenbildenden Hautbereichen.^33^

Bei Wundinfektionen ist häufig eine wiederholte Antibiotikabehandlung erforderlich. Diese kann jedoch zu einer selektiven Dysbiose des kutanen Mikrobioms führen und die Entstehung multiresistenter Keime wie Methicillin‐resistenter *S. aureus* (MRSA) begünstigen.^34^, ^35^

Das Hautmikrobiom unseres JEB‐Patienten unterschied sich je nach Hautstatus. Das Mikrobiom der transgenen Haut lag in der Zusammensetzung zwischen der blasenbildenden und der nicht‐blasenbildenden Haut. Die blasenbildende Haut zeigte einen höheren Anteil von *S. aureus* im Vergleich zur nicht‐blasenbildenden und transgenen Haut, während transgene und nicht‐blasenbildende Haut sich ähnelten. Da sich das Mikrobiom je nach Körperregion unterscheidet, war es bei unserem Patienten nicht trivial, Proben von allen drei Gewebetypen (blasenbildend, nicht‐blasenbildend und transgen) zu entnehmen, was die Anzahl möglicher Testbereiche reduzierte.

Wir konnten ein deutlich unterschiedliches globales Mikrobiom des untersuchten JEB‐Patienten nach Transplantation transgener Haut im Vergleich zu gesunden Kontrollpersonen und AD‐Patienten nachweisen. Die absoluten Bakterienzellzahlen in der blasenbildenden Haut entsprachen dabei den in der ProRaD‐Studie bei AD‐Patienten beobachteten Werten.

Trotz dieser Dysbiose blieben die transgenen Bereiche stabil und zeigten keine klinischen Anzeichen von Ekzemen oder Blasenbildung. Dies bestätigt eine genetische Stabilität der mit Vektor‐transduzierten Hautbereiche gegen ein Wiederauftreten von Blasenbildung sowie eine Resistenz gegenüber mikrobieller Dysbiose.

Die Hauptlimitation dieser Studie ist die geringe Stichprobengröße, da unser Patient bislang der einzige ist, der die oben beschriebene Behandlung erhalten hat. Die Probenahme an Bereichen, in denen alle drei Gewebetypen (blasenbildend, nicht‐blasenbildend und transgen) vorhanden waren, schränkte die Anzahl möglicher Testorte ein, wodurch die Probenanzahl klein bleibt. Daher ist keine statistische Analyse für Körperregionen möglich. Um unsere Daten zu validieren, wurden unabhängige Replikate durchgeführt, die zuverlässige Ergebnisse zeigten. Dennoch sind intraindividuelle Unterschiede sowie Umwelteinflüsse möglich.

Zukünftige Untersuchungen sollten sich auf Follow‐up‐Studien des Mikrobioms des transplantierten JEB‐Patienten konzentrieren. Diese könnten klären, ob das Mikrobiom beispielsweise hormonellen Veränderungen während der Pubertät oder Adoleszenz unterliegt. Darüber hinaus sollte die Untersuchung des Umfelds auf die Familie unseres Patienten ausgeweitet werden, um zu prüfen, ob Familienangehörige mit demselben *S.‐aureus*‐Stamm kolonisiert sind wie der Patient.

## FÖRDERUNG

Diese Studie wurde im Rahmen der *Prospective longitudinal study to investigate the remission phase in patients with atopic dermatitis* vom *Christine Kühne‐Center for Allergy Research and Education* (CK‐CARE), Davos, Schweiz, gefördert. Für die Durchführung der vorliegenden Untersuchung wurden keine weiteren Fördermittel eingeworben.

## DANKSAGUNG

Open access Veröffentlichung ermöglicht und organisiert durch Projekt DEAL.

## INTERESSENKONFLIKT

Keiner.

## Supporting information



Supplementary information
