# Supplementary material for: Analyse des Hautmikrobioms eines an junktionaler Epidermolysis bullosa erkrankten Patienten nach Behandlung mit genetisch modifizierten Stammzellen
Source: J Dtsch Dermatol Ges. 2025 Sep 15;23(9):1084–93. [Article in German] doi: 10.1111/ddg.15776_g (PMC12435134; doi:10.1111/ddg.15776_g)
Supplement: Supplementary file 1 — Supplementary information [file DDG-23-1084-s001.docx]

**Online ergänzende Abbildungen**

**Online ergänzende Abbildung A1.**

**
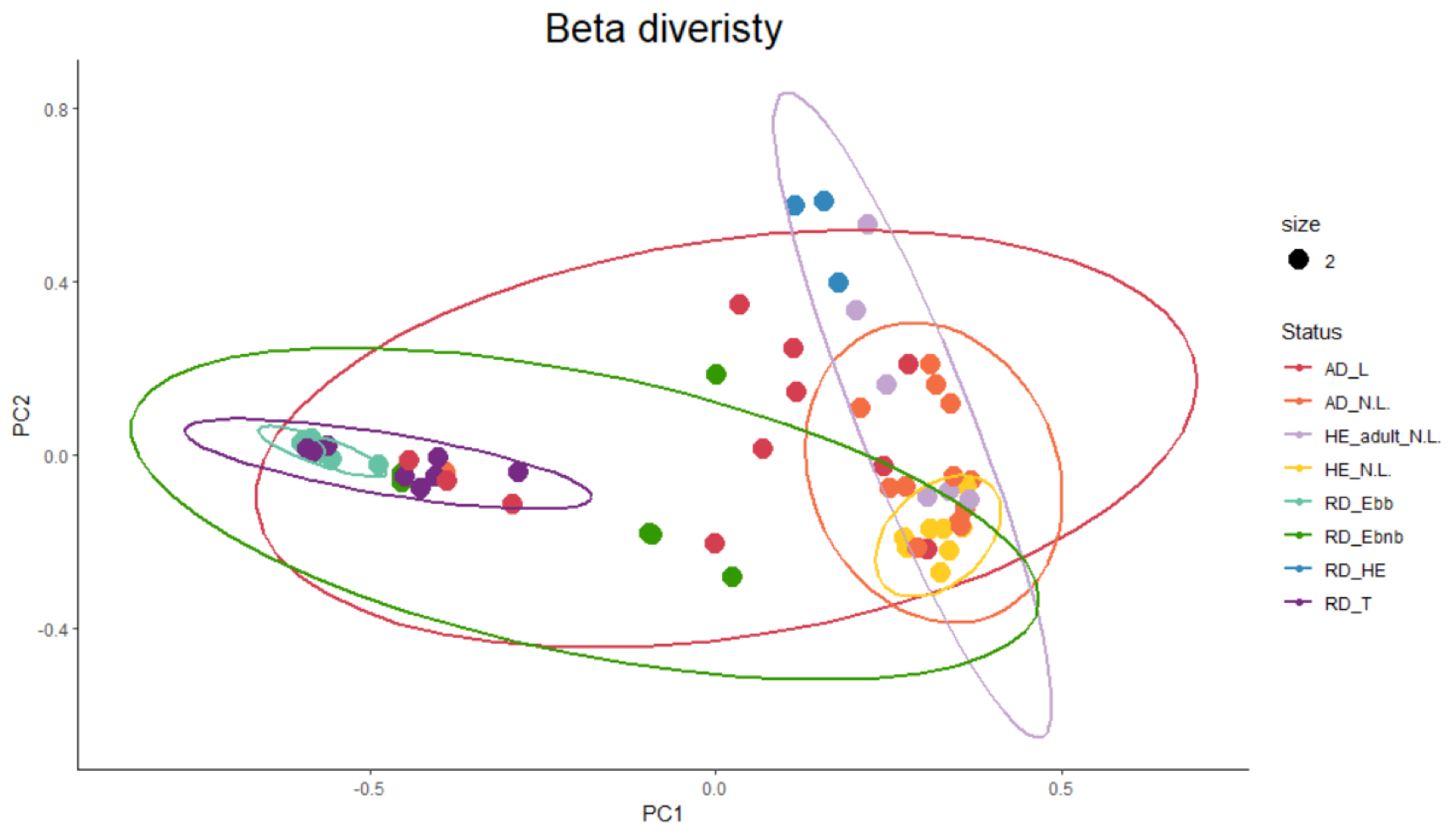

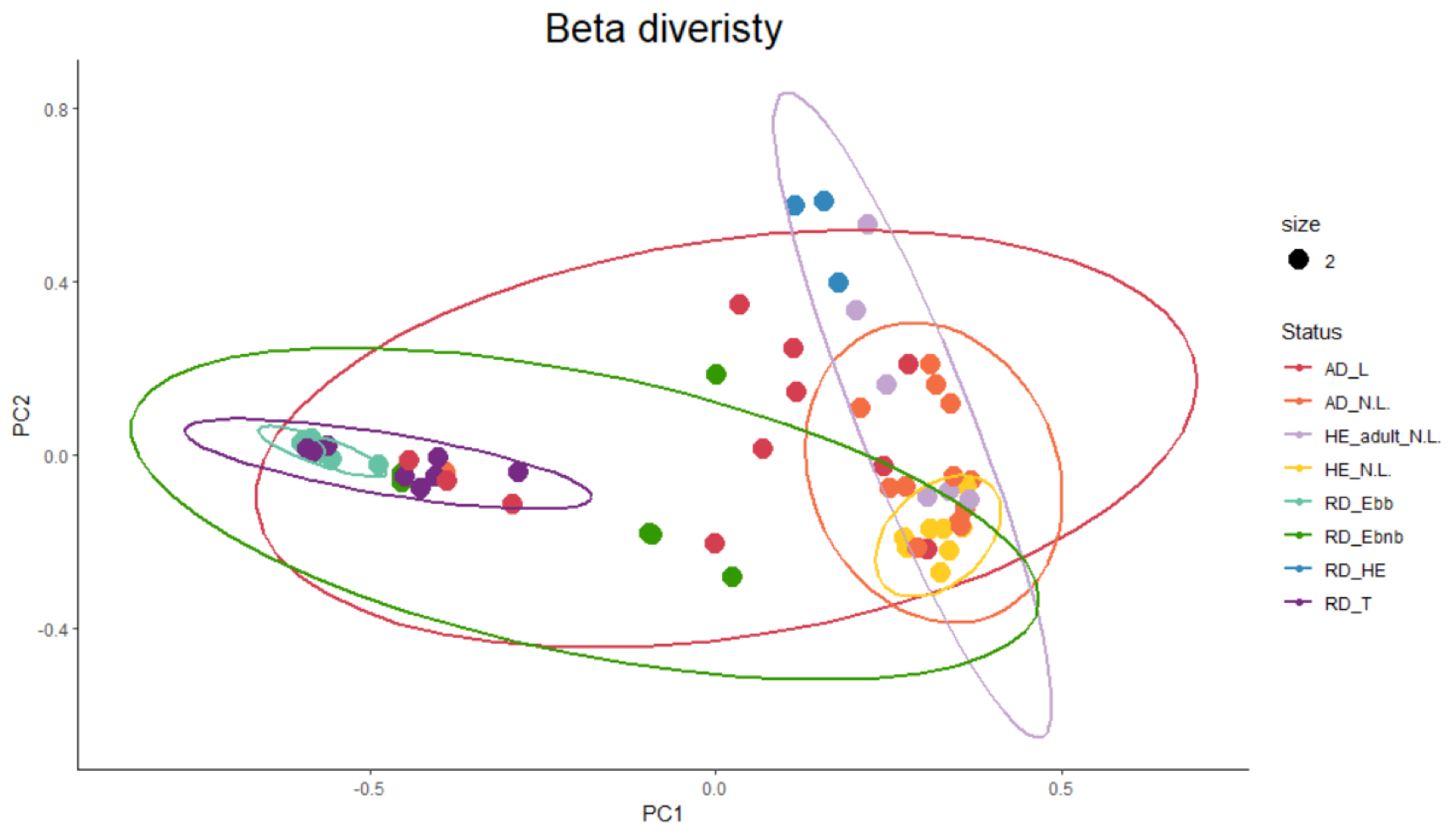
**

***Online ergänzende Abbildung A1: Globales Hautmikrobiom des JEB-Patienten, AD und HE****. Das globale Mikrobiom gesunder Individuen, Patienten mit atopischer Dermatitis und dem JEB-Patienten. Das globale Hautmikrobiom, dargestellt durch eine Hauptkoordinatenanalyse (PCoA) basierend auf der Bray-Curtis-Distanz, zeigte ein deutlich unterschiedliches Mikrobiom im Vergleich zu gesunden Kontrollen und AD-Patienten. Da der gesunde Erwachsene aus dem Epidermolysis bullosa-Datensatz und aus dem ProRaD-Datensatz zusammen geclustert ist, war die Methode zur Kombination der Datensätze geeignet. HE = gesunde Kontrolle, AD = atopische Dermatitis, RD = seltene Erkrankung/Epidermolysis bullosa, NL = nicht-lesionale Haut, L = lesionale Haut, Ebb = blasenbildende Haut bei junktionaler Epidermolysis bullosa, Ebnb = nicht-blasenbildende Haut bei junktionaler Epidermolysis bullosa, T = transgene Haut bei junktionaler Epidermolysis bullosa.*

**Online ergänzende Abbildung A2.**


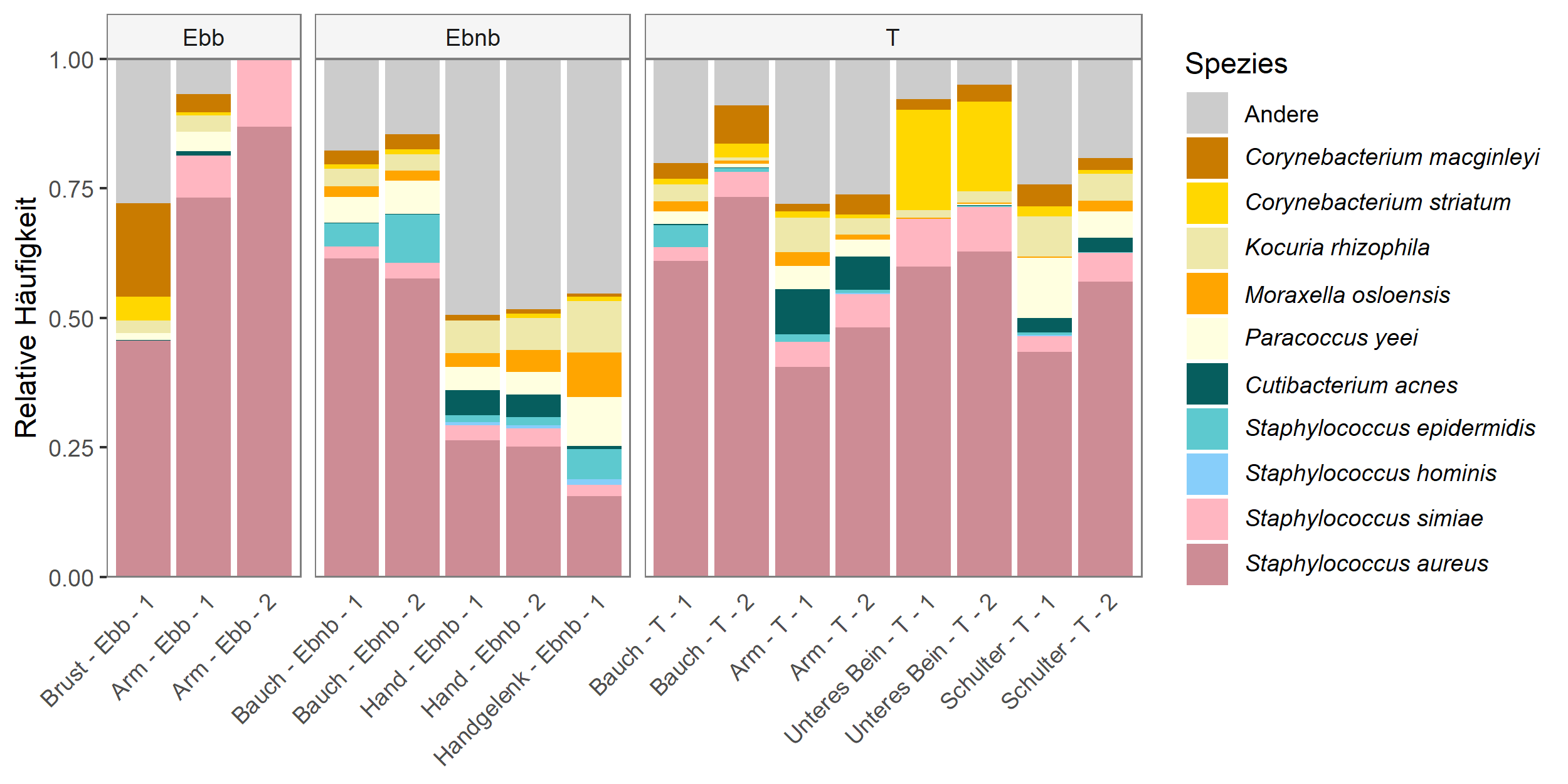


***Online ergänzende Abbildung A2:*** ***Mikrobiom-Taxonomie der blasenbildenden, nicht-blasenbildenden und transgenen Haut des JEB-Patienten.*** *Die transgene Haut weist ein ähnlich hohes Vorkommen von S. aureus auf wie die blasenbildende Haut. Dargestellt sind individuelle Proben pro Standort. Ebb = blasenbildende Haut bei junktionaler Epidermolysis bullosa, Ebnb = nicht-blasenbildende Haut bei junktionaler Epidermolysis bullosa, T = transgene Haut bei junktionaler Epidermolysis bullosa.*

**Online ergänzende Abbildung A3.**


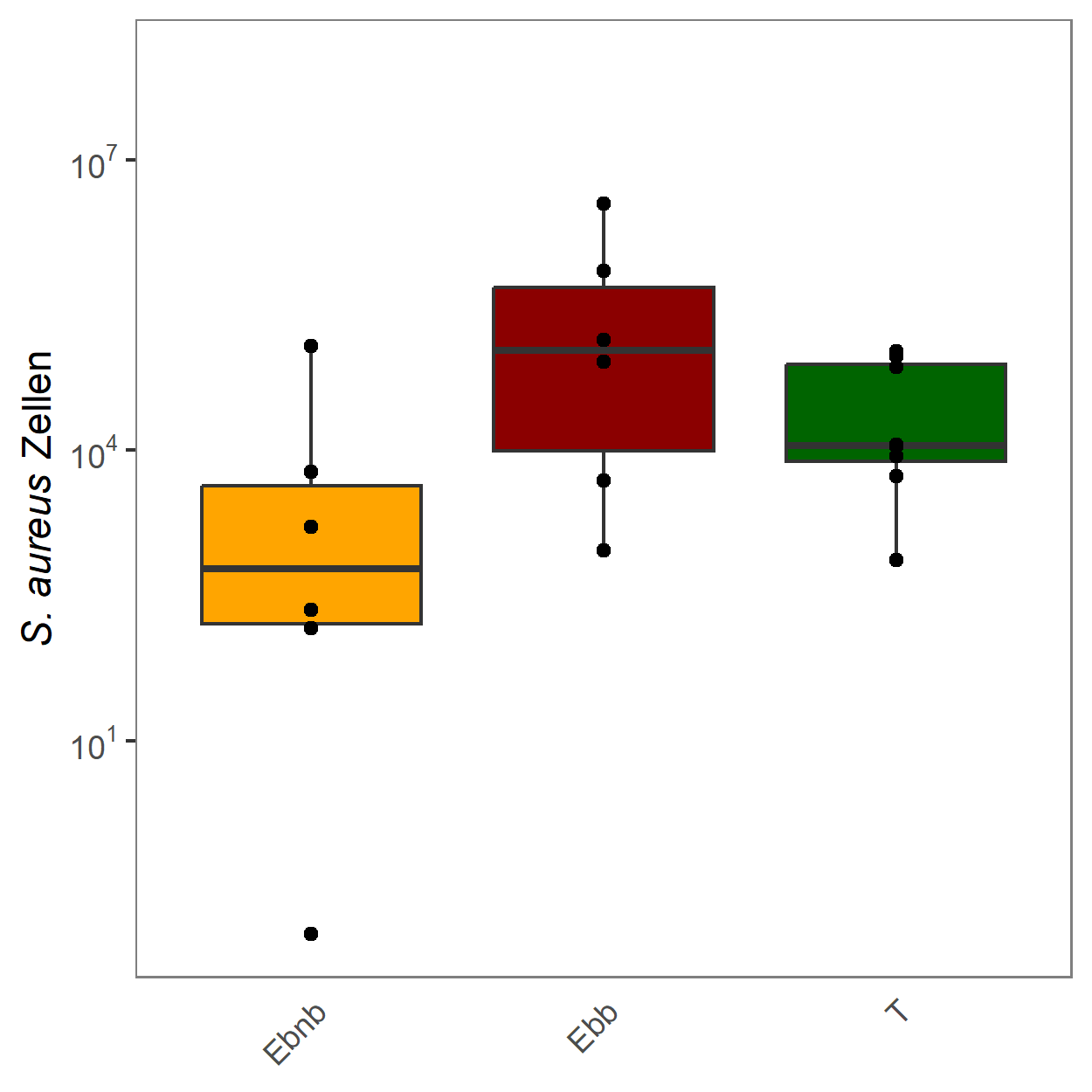


***Online ergänzende Abbildung A3: S. aureus Zellzahlen des JEB-Patienten.*** *Die absoluten Zellzahlen von S. aureus, gemessen mittels qPCR, sind in der blasenbildenden Haut am höchsten und in der nicht-blasenbildenden Haut am niedrigsten. Ebb = blasenbildende Haut bei junktionaler Epidermolysis bullosa, Ebnb = nicht-blasenbildende Haut bei junktionaler Epidermolysis bullosa, T = transgene Haut bei junktionaler Epidermolysis bullosa.*

**Online ergänzende Abbildung A4.**


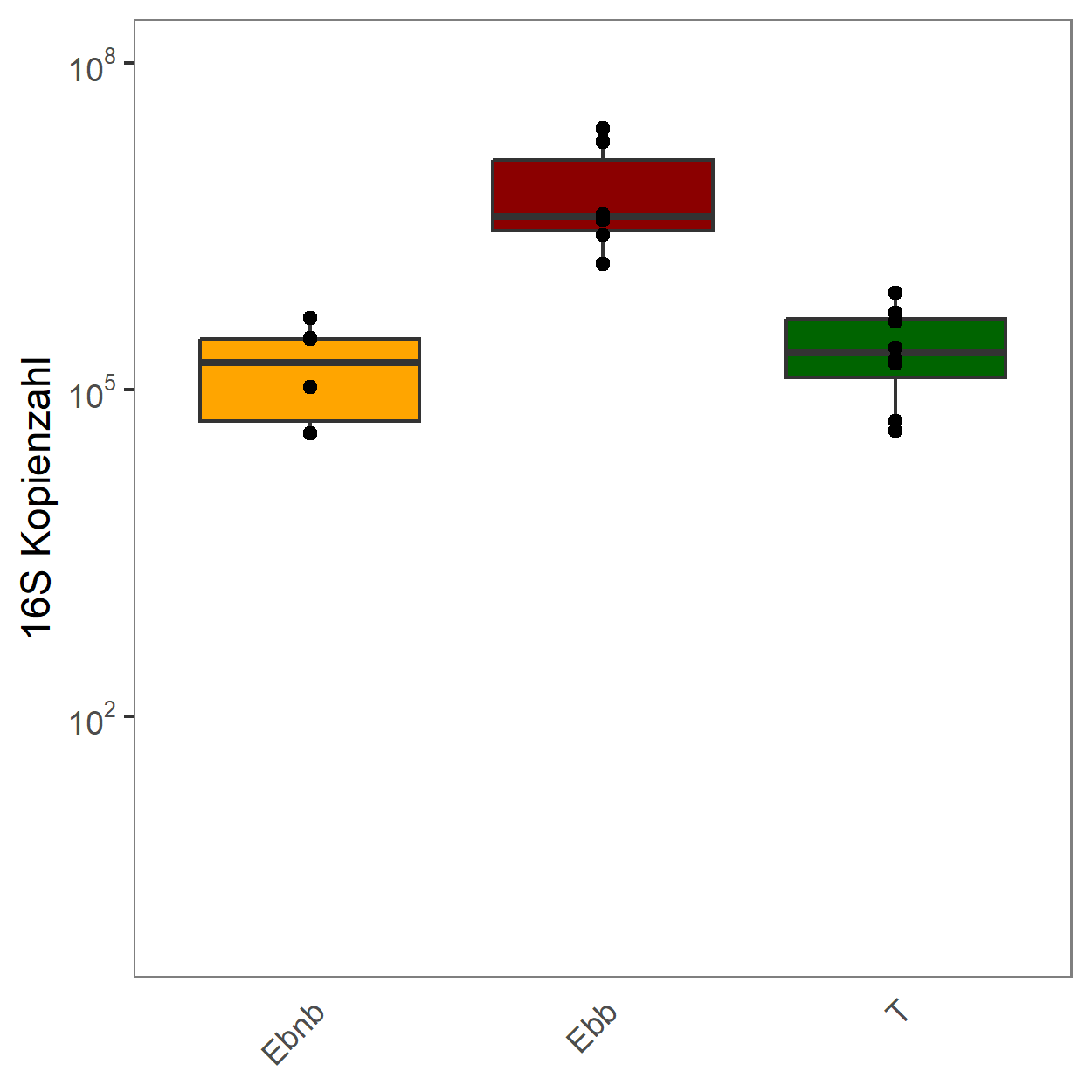


***Online ergänzende Abbildung A4: Gesamte bakterielle Last des JEB-Patienten.*** *Die absolute bakterielle Last, die mittels qPCR unter Verwendung von 16S-Kopienzahlen als Proxy nachgewiesen wurde, ist in der blasenbildenden Haut am höchsten, während die nicht-blasenbildende und die transgene Haut vergleichbare Werte aufweisen. Ebb = blasenbildende Haut bei junktionaler Epidermolysis bullosa, Ebnb = nicht-blasenbildende Haut bei junktionaler Epidermolysis bullosa, T = transgene Haut bei junktionaler Epidermolysis bullosa.*
